# Supplementary material for: Identification of genes supporting cold resistance of mammalian cells: lessons from a hibernator
Source: Cell Death Dis. 2024 Sep 19;15(9):685. doi: 10.1038/s41419-024-07059-w (PMC11413375; doi:10.1038/s41419-024-07059-w)
Supplement: Supplementary file 1 — Supplementary Information [file 41419_2024_7059_MOESM1_ESM.pdf]

## Supplemental figure legends

**Fig. S1. Metabolome analysis revealed glutathione synthesis pathway is retained in hamster cells under cold and nutrient-deprived condition**

**a** Amount of metabolites related to glutathione synthesis before or under cold temperature (4˚C) and nutrient deprivation. **b** Amount of nucleotides before or under cold temperature (4˚C) and nutrient deprivation. **c** Amount of amino acids before or under cold temperature (4˚C) and nutrient deprivation. **d** Amount of NAD, NADH, NADPH before or under cold temperature (4˚C) and nutrient deprivation. **e** Hierarchical clustering of metabolome profiles. Representative metabolites are shown. One-way ANOVA with the Dunnett's multiple comparison test compared to day0 in C-F, *$p < 0.05$, **$p < 0.01$, ***$p < 0.001$, ****$p < 0.0001$

**Fig. S2 Expression levels of Gpx4 in HT1080, HapT1 and liver tissues of mice and hamsters**

**a** Immunoblot of Gpx4 and β-Actin proteins in HT1080 and HapT1. **b** Immunoblot of Gpx4 and β-Actin proteins in mouse and hamster livers (N = 4). **c** Immunoblot of Gpx4 and β-Actin proteins in HT1080 and HapT1 under cold temperature (4˚C) for indicated period. Signals were quantified and represented as bar plot in the right panel. **d** Immunoblot of Gpx4 and β-Actin proteins in livers of hamsters in the states of non-hibernation (room temperature, RT), periodic arousal (PA) and deep torpor (DT) (N = 3 or 4). Signals were quantified and represented as box plot in the right panel.

**Fig. S3 Biopterin and CoQ reduction pathways are required for short-term cold-resistance under Gpx4 dysfunction in hamster cells, related to Fig. 5**

**a - c** The proportion of dead cells during cold culture (4ºC) culture in the each HapT1 cell population infected with lentivirus expressing SpCas9 and non-targeting sgRNA (sgNT) or sgRNA for FSP1 (**a**) or Dhodh (**b**) or Gch1 (**c**) in the presence or absence of 6μM ML210 (One-way ANOVA with the Tukey's multiple comparison test, $p < 0.05$). **d** Assessment of knock-out efficiency of Spr in HapT1 cells. Immunoblots of Spr and β-Actin proteins in HapT1 cell populations infected with lentivirus vectors expressing SpCas9 and sgNT or sgRNA targeting Spr. **e** Assessment of knock-out efficiency of Pts in HapT1 cells. Quantitative RT-PCR of Spr and β-Actin mRNA in HapT1 cell populations infected with lentivirus vectors expressing SpCas9 and sgNT or sgRNA targeting Pts. Note that Pts mRNA was decreased in the cells introduced with sgPts presumably through nonsense mediated RNA decay pathway. **f** The proportion of dead

cells after two-day cold culture in HapT1 infected with lentivirus expressing SpCas9 and sgNT or sgRNA for Spr or Pts and treated with 2μM RSL3 or 6μM ML210 determined by LDH assay (One-way ANOVA with the Dunnett's multiple comparison test, $**p < 0.01$, $***p < 0.001$). **g** The proportion of dead cells during cold culture in HapT1 infected with lentivirus expressing SpCas9 and sgNT or sgRNA for Gch1 with or without 100μM BH2 and 4μM methotrexate (MTX) determined by LDH assay (N = 3 wells).

Fig. S1

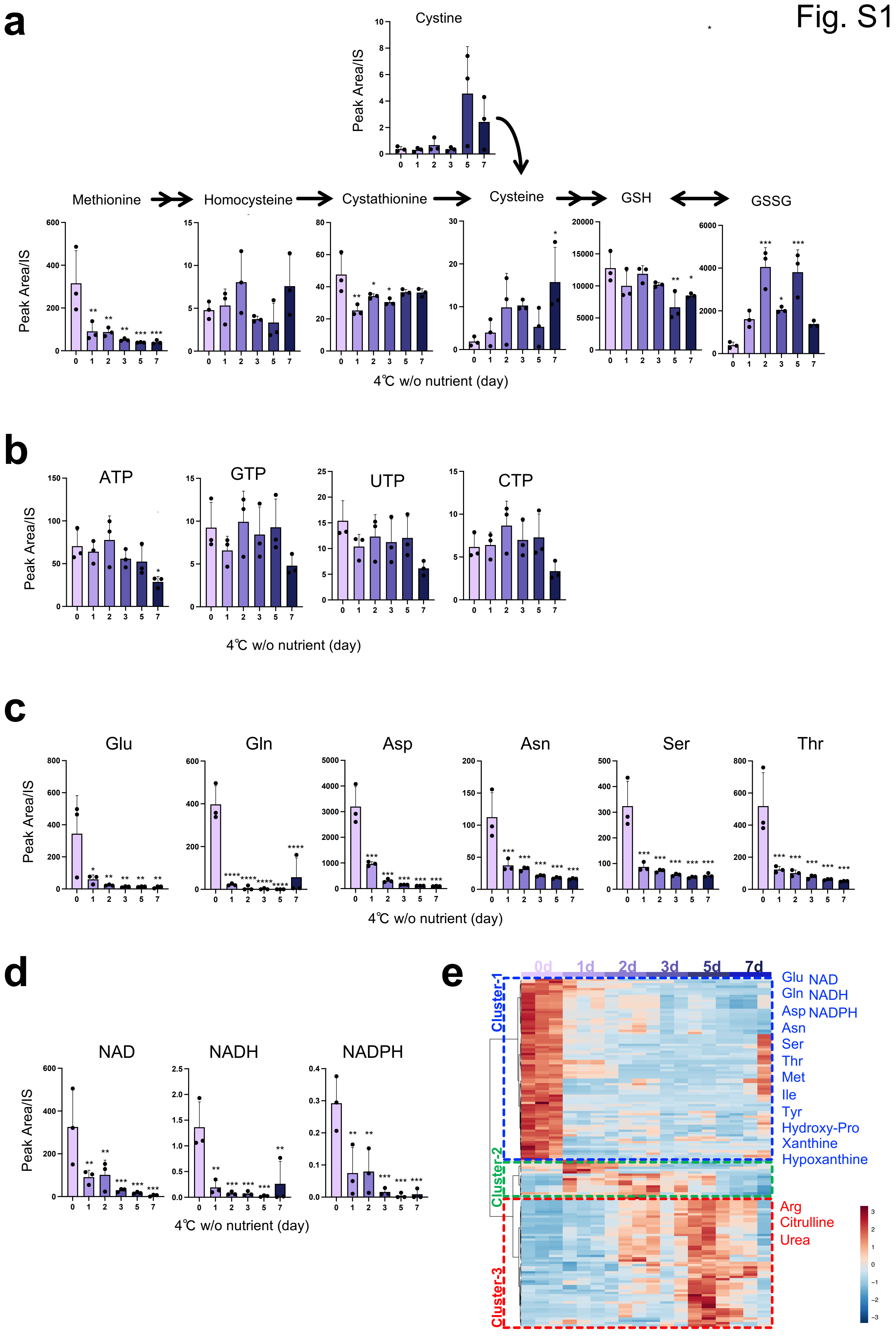

a
Cystine
Peak Area/IS
Methionine
Homocysteine
Cystathionine
Cysteine
GSH
GSSG
4℃ w/o nutrient (day)
b
ATP
GTP
UTP
CTP
Peak Area/IS
4℃ w/o nutrient (day)
c
Glu
Gln
Asp
Asn
Ser
Thr
Peak Area/IS
4℃ w/o nutrient (day)
d
NAD
NADH
NADPH
Peak Area/IS
4℃ w/o nutrient (day)
e
0d
1d
2d
3d
5d
7d
Cluster-1
Cluster-2
Cluster-3
Glu
Gln
Asp
Asn
Ser
Thr
Met
Ile
Tyr
Hydroxy-Pro
Xanthine
Hypoxanthine
NAD
NADH
NADPH
Arg
Citrulline
Urea

Fig.S2

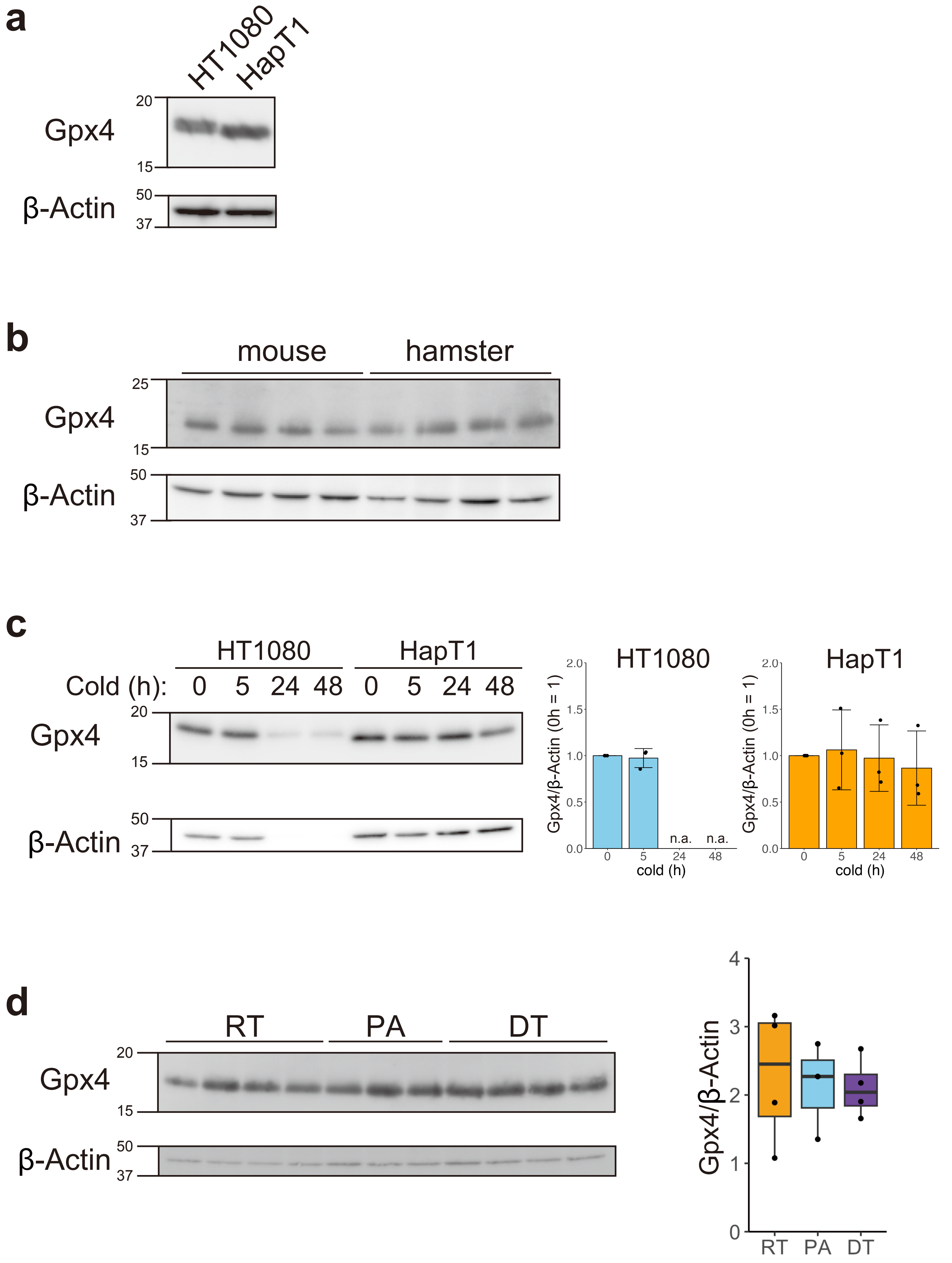

a
HT1080
HapT1
Gpx4
20
15
β-Actin
50
37
b
mouse
hamster
Gpx4
25
15
β-Actin
50
37
c
HT1080
HapT1
Cold (h): 0 5 24 48 0 5 24 48
Gpx4
20
15
β-Actin
50
37
HT1080
Gpx4/β-Actin (0h = 1)
n.a.
n.a.
cold (h)
HapT1
Gpx4/β-Actin (0h = 1)
cold (h)
d
RT
PA
DT
Gpx4
20
15
β-Actin
50
37
Gpx4/β-Actin
RT PA DT

Fig. S3

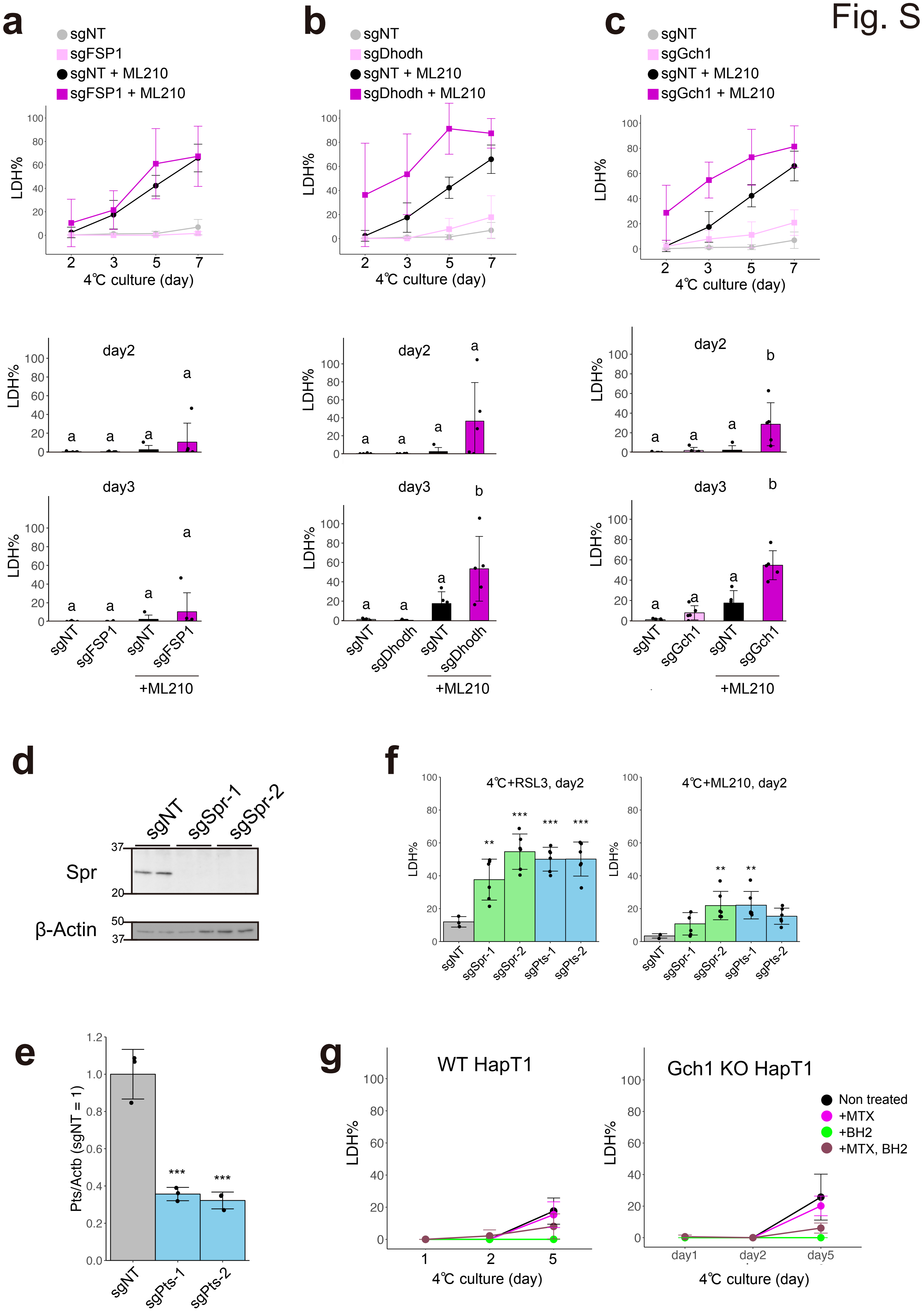
a
sgNT
sgFSP1
sgNT + ML210
sgFSP1 + ML210
LDH%
4℃ culture (day)
day2
day3
sgNT
sgFSP1
sgNT
sgFSP1
+ML210
b
sgNT
sgDhodh
sgNT + ML210
sgDhodh + ML210
sgDhodh
c
sgNT
sgGch1
sgNT + ML210
sgGch1 + ML210
sgGch1
d
sgNT
sgSpr-1
sgSpr-2
Spr
β-Actin
e
Pts/Actb (sgNT = 1)
sgPts-1
sgPts-2
f
4℃+RSL3, day2
4℃+ML210, day2
g
WT HapT1
Gch1 KO HapT1
Non treated
+MTX
+BH2
+MTX, BH2
day1
day2
day5
