# Supplementary figures and images for: Identification of genes supporting cold resistance of mammalian cells: lessons from a hibernator
Source: Cell Death Dis. 2024 Sep 19;15(9):685. doi: 10.1038/s41419-024-07059-w (PMC11413375; doi:10.1038/s41419-024-07059-w)

**Fig. 2a**

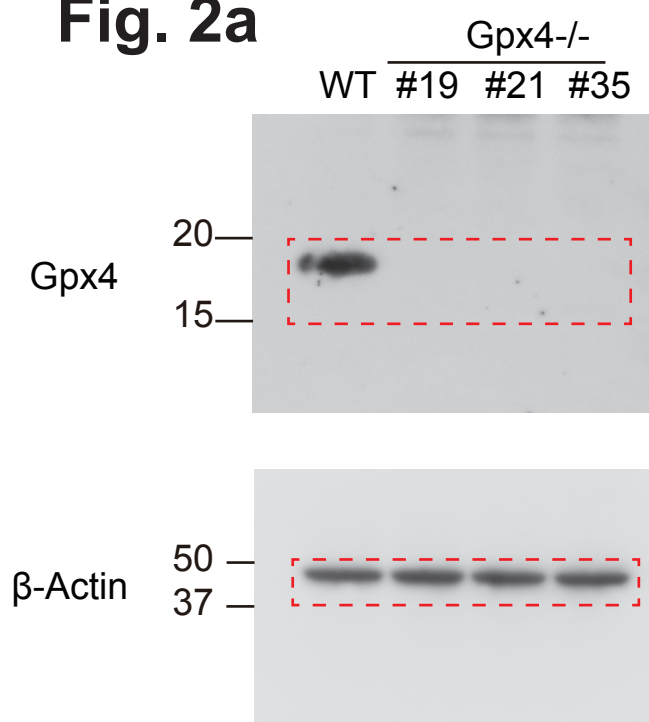

**Fig. 3a**

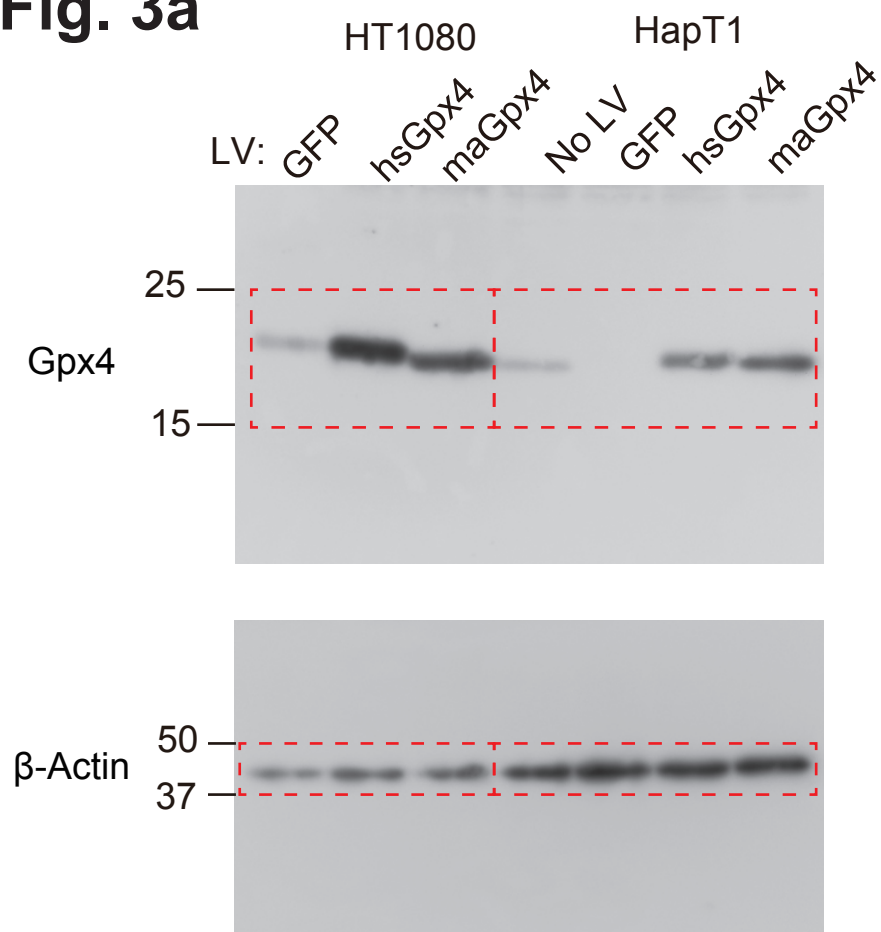

Fig. 4a

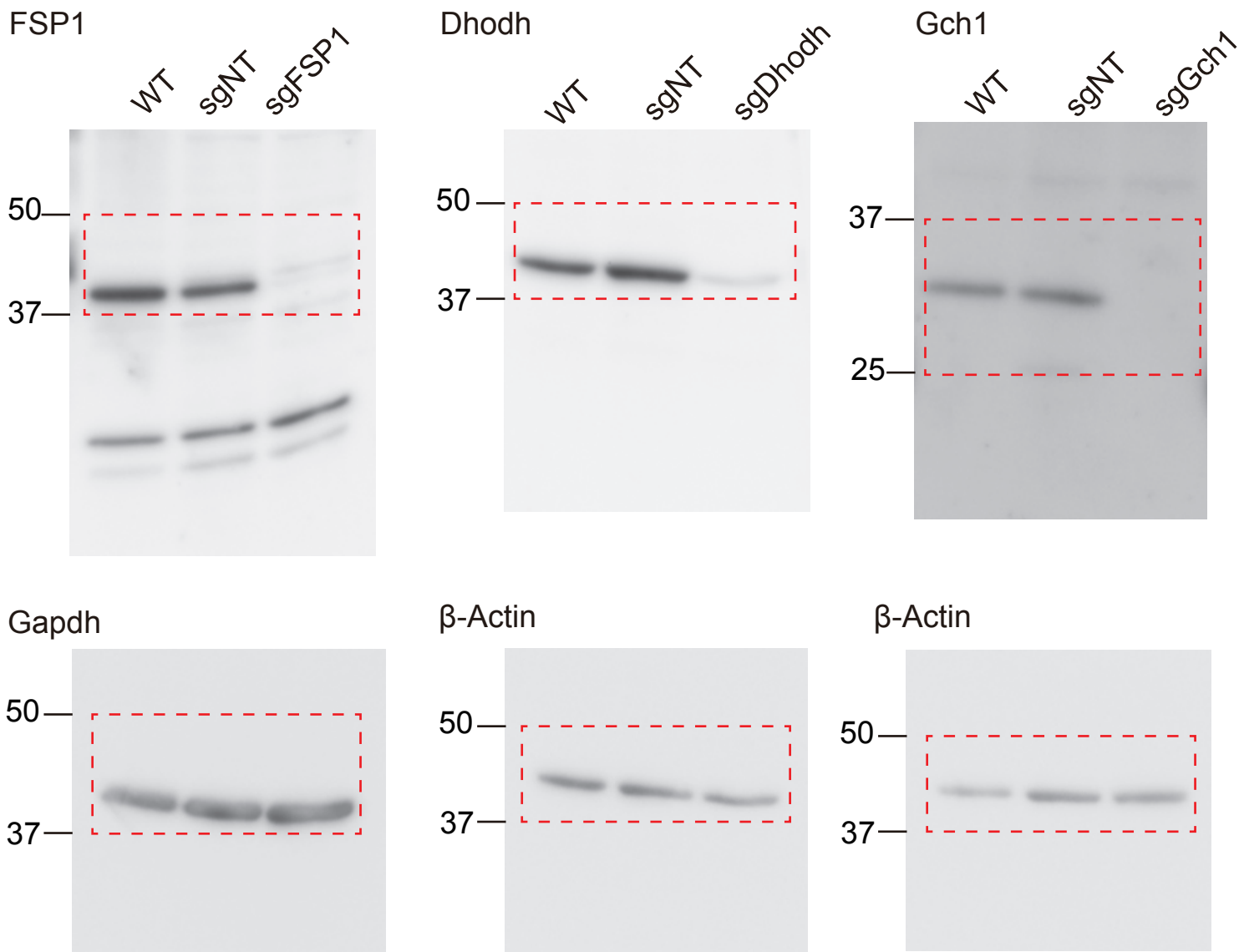

**Fig. 5g**

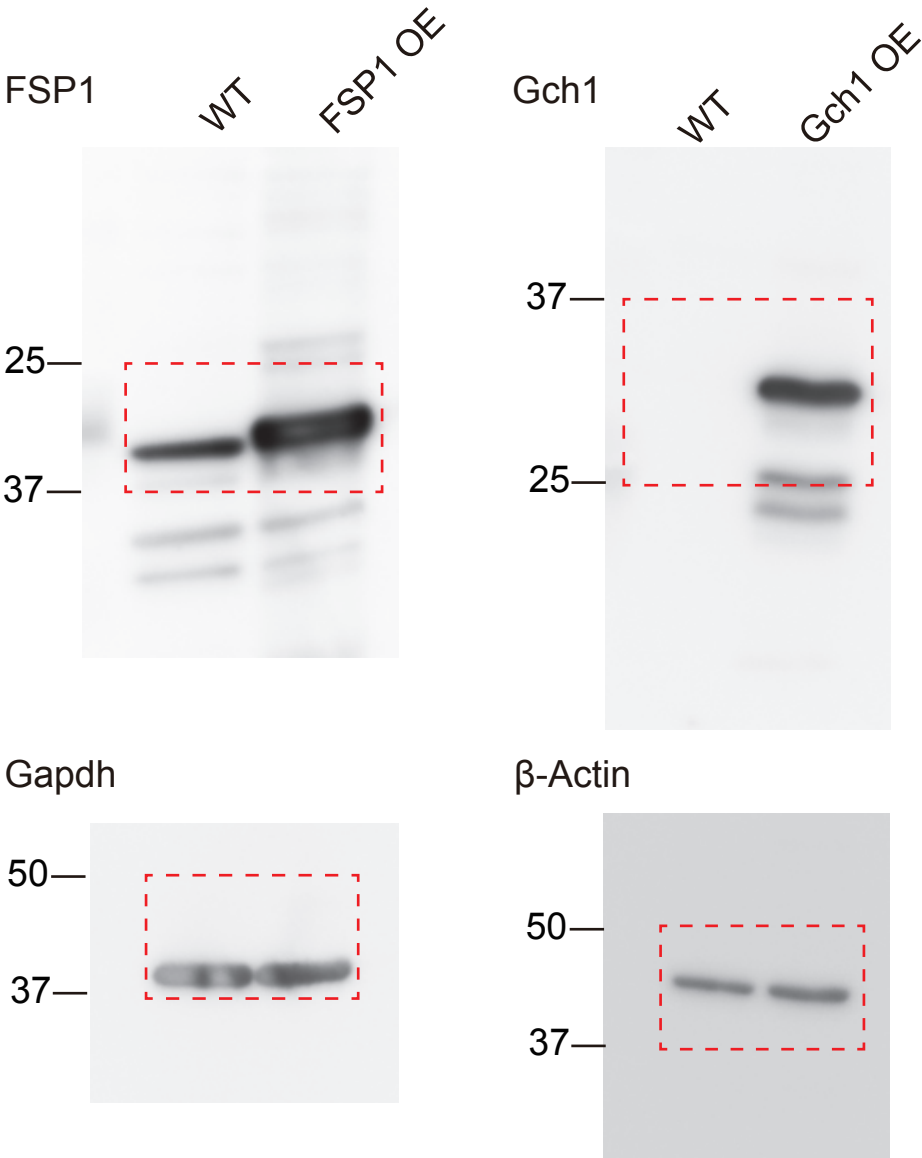

**Fig. 6b**

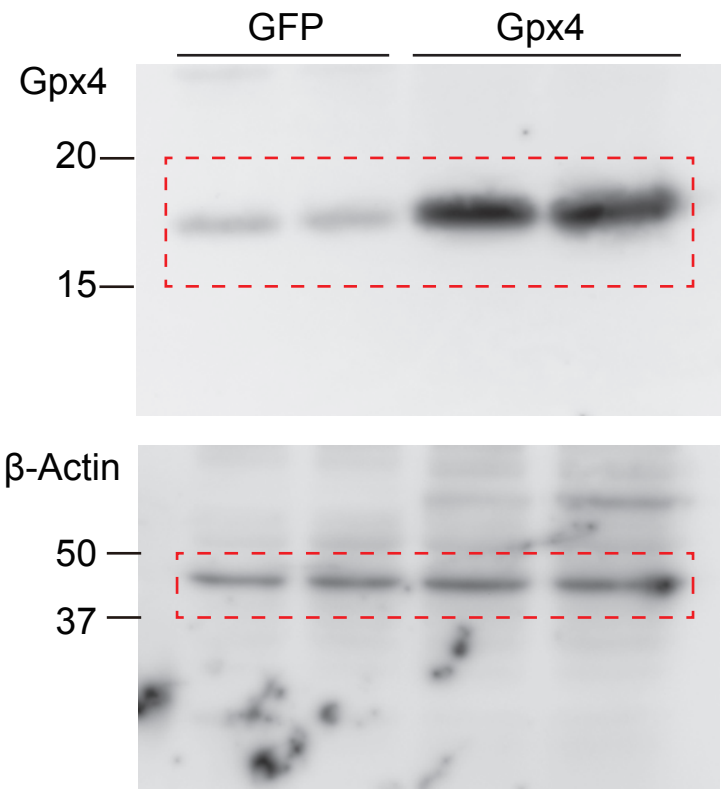

**Fig. S2a**

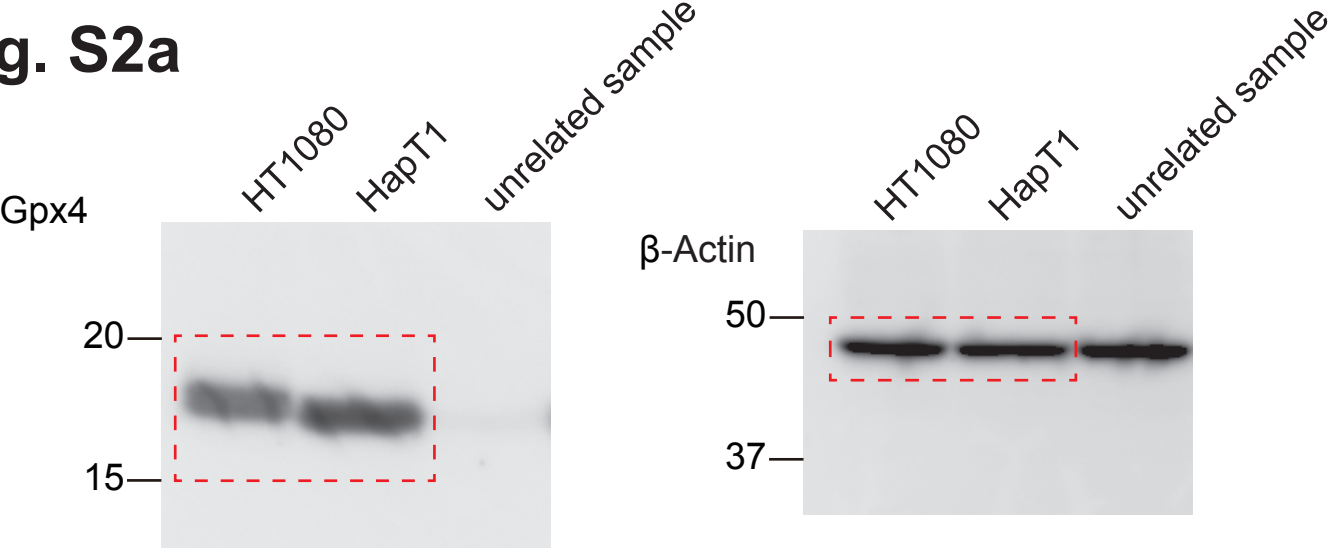

**Fig. S2b**

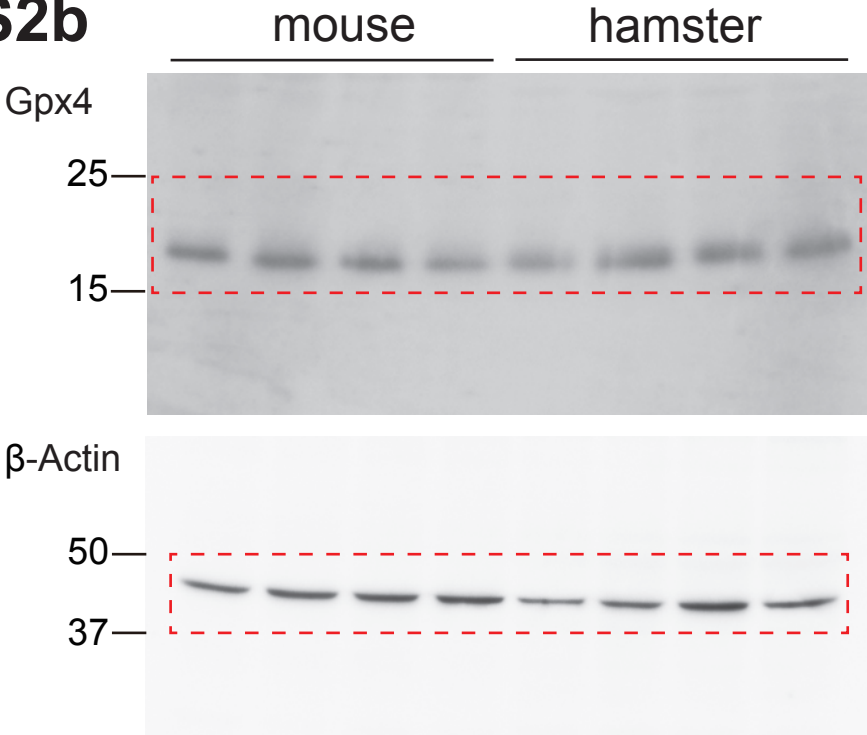

**Fig. S2c**

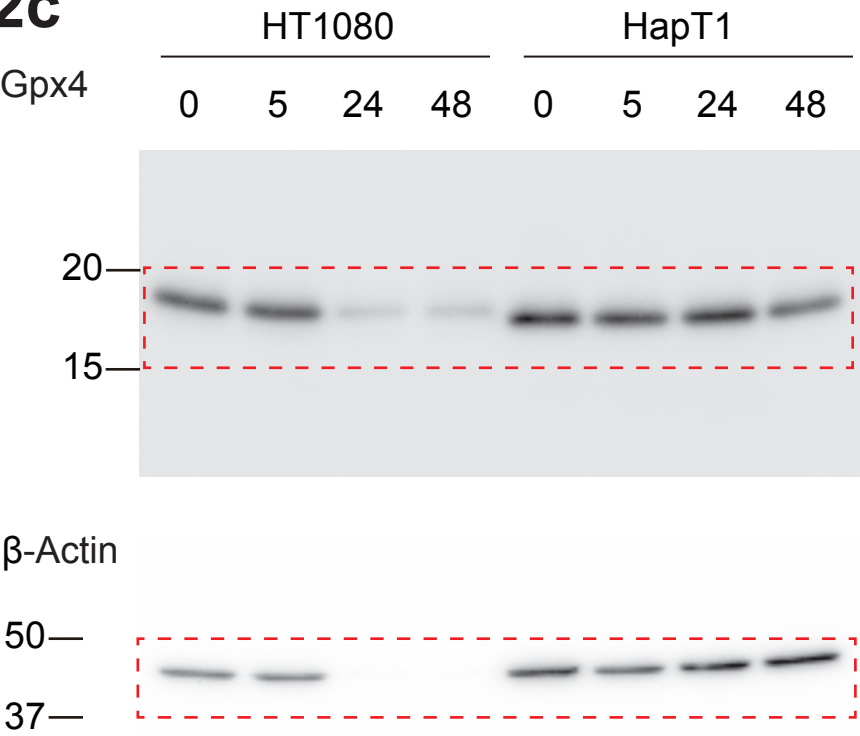

**Fig. S2d**

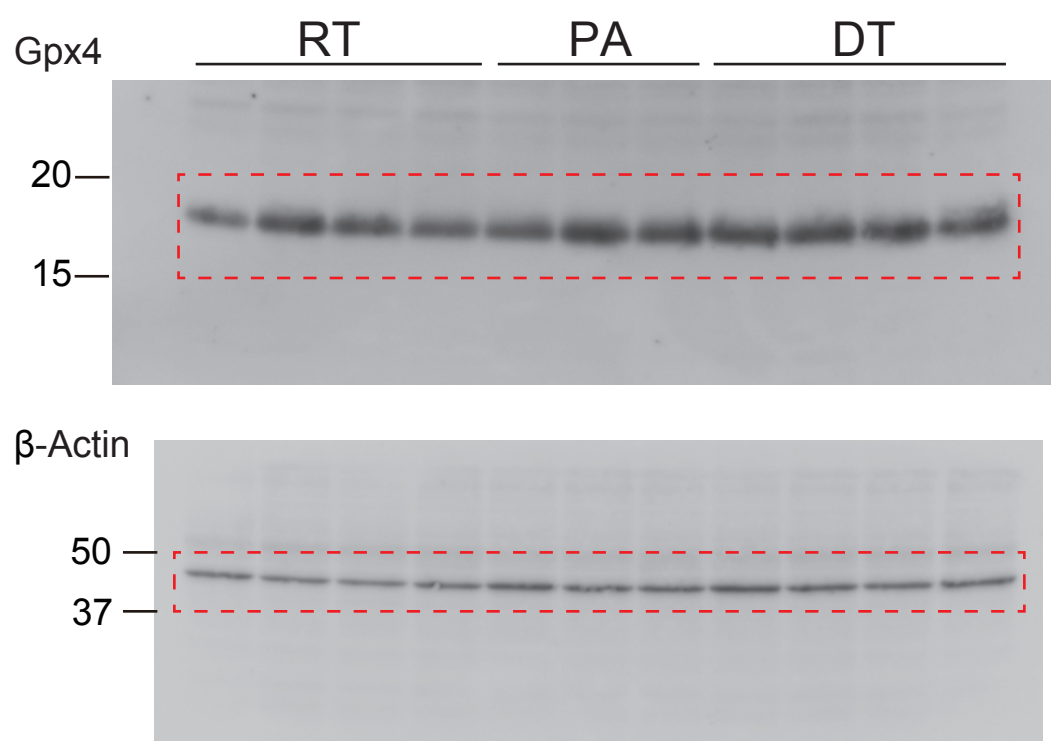

Supplement: Supplementary file 3 — Uncropped Western Figure [file 41419_2024_7059_MOESM3_ESM.pdf]
